# Validation of the i-Tracker Drug and Total Anti-Drug Antibody CLIA Assays on IDS-iSYS for Therapeutic Drug Monitoring in Adalimumab- and Infliximab-Treated Patients

**DOI:** 10.3390/diagnostics15192447

**Published:** 2025-09-25

**Authors:** Akpedje Serena Dossou, Serena Kang, Tahira Kalhoro, Eduardo Castro-Echeverry, Nathan C. Horton

**Affiliations:** Sonic Reference Laboratory, Austin, TX 78728, USA

**Keywords:** TDM, Adalimumab, Infliximab, ADAs, i-Tracker, total anti-Adalimumab, total anti-Infliximab, drug tolerance

## Abstract

**Background/Objectives**: Adalimumab and Infliximab are biologics used to treat autoimmune diseases. Monitoring drug and anti-drug antibody (ADA) levels in patients helps optimize treatment. However, current quantitation methodologies for drug and total (free and drug-bound) ADAs often involve multi-step workflows. Automated systems can streamline the process. The i-Tracker chemiluminescent immunoassays (CLIA) are cartridge-based kits for quantifying serum levels of drugs such as Adalimumab, Infliximab, and associated ADAs. Herein, we aimed to establish performance characteristics of the i-Tracker Adalimumab, Infliximab, and total ADAs in serum on the random-access analyzer IDS-iSYS and to compare patient results with an electrochemiluminescent immunoassay (ECLIA)-based reference method. **Methods**: Remnant serum specimens, calibration material, or spiked serum were used to evaluate assay linearity, precision, functional sensitivity, and accuracy on the IDS-iSYS analyzer and to perform the method comparison. **Results**: The assays displayed linearity, accuracy, and up to 8% imprecision across clinically relevant analyte ranges. Compared to the reference method, the drug assays exhibited a strong linear fit (correlation coefficient > 0.95) with <±1.0 µg/mL mean bias. The total anti-Adalimumab assay demonstrated over 85% qualitative agreement. The total anti-Infliximab assay, however, showed higher detection rate of ADAs in Infliximab-treated patient specimens, yielding < 60% negative agreement with the reference method. Although i-Tracker total ADA assays exhibited drug sensitivity, they still detected ADAs in supratherapeutic drug concentrations. **Conclusions**: The i-Tracker assays demonstrated robust analytical performance, suggesting potential for clinical application. The method comparison underscored functional differences with the reference method, an important consideration when transitioning assay formats for monitoring Adalimumab- and Infliximab-treated patients.

## 1. Introduction

While the etiology of autoimmune diseases is not always clear, it is recognized that aberrantly produced pro-inflammatory cytokines are important actors in the pathogenesis and pathophysiology of these disorders [1]. Tumor necrosis factor alpha (TNFα) is one of the major pro-inflammatory cytokines often associated with chronic inflammatory conditions, including gastrointestinal, rheumatologic, and dermatologic disorders. By interacting with its receptors at the cell surface, TNFα elicits signal transduction that promotes inflammation, cell death, and ultimately connective tissue degradation and epithelial tissue damage [2,3,4]. Hence, TNFα signaling constitutes a critical therapeutic target. Anti-TNFα strategies have benefited from advances in therapeutic monoclonal antibody immunology, and anti-TNFα antibodies are now part of the clinical management portfolio of autoimmune inflammatory conditions [5].

Adalimumab (ADM) and Infliximab (IFX) are, respectively, a fully human and a chimeric mouse/human recombinant anti-TNFα monoclonal IgG1 antibody. Both drugs are approved by the Food and Drug Administration (FDA) and widely used as first-line biologics to treat various autoimmune diseases. These diseases include Crohn’s disease, ulcerative colitis, rheumatoid arthritis (RA), ulcerative ankylosing spondylitis, psoriatic arthritis, and plaque psoriasis [6,7,8]. ADM and IFX exert their anti-inflammatory effects in patients by binding to both soluble and transmembrane TNFα. This interaction disrupts the inflammatory signaling cascade and can provoke the apoptosis of TNFα-expressing immune cells [9,10]. Both drugs have proven successful in achieving and maintaining remission in some patients, and their trough levels (i.e., the circulating drug level just before the next injection) have been demonstrated to correlate with clinical efficiency [11,12]. However, trough levels are influenced by many factors including disease activity, pharmacogenetic factors, dosage, frequency of injections, and the development of anti-drug antibodies (ADAs). Like many biologics, ADM and IFX are prone to immunogenicity, and ADAs are often responsible for secondary treatment failure in the clinics [13,14]. Along with generating adverse reactions with drug infusions, the ADAs can neutralize drugs, impede their activity, or cause their early clearance. The ensuing subtherapeutic levels of the drug result in low response to treatment [15].

Therapeutic drug monitoring (TDM)—or the monitoring of circulating levels of the drug and associated ADAs in patient serum—provides valuable information to optimize biologics-based treatment of inflammatory autoimmune diseases. Based on TDM results, drug dosage can be adjusted to maintain therapeutic levels. Additionally, with TDM, clinicians can gain insight into treatment failure and pivot if needed to implement a more beneficial treatment strategy for the patient, i.e., switch to another anti-TNFα biologic, a different class of biologics, or a different line of treatment altogether [16,17,18]. Various procedural approaches with their own benefits and limitations can be used for TDM in patients. Methodologies available for both drug and ADA quantification include enzyme-linked immunosorbent assay (ELISA), radioimmunoassay (RIA), homogeneous mobility shift assay (HMSA), functional cell-based reporter gene assay, and chemiluminescent immunoassay (CLIA)-based methodology [19,20]. While mass spectrometry (MS)-based methods are not commonly used for ADA quantification due to the complex biochemistry of ADAs and processing challenges [21,22], liquid chromatography and tandem MS (LC-MS/MS) methods can be used for drug quantification in patient samples [23,24]. Rapid fluorescence-based point-of-care-testing methodologies have also emerged for drug quantification [25,26]. However, with the ease of standardization they offer for the panel composed of the drug and ADA quantification, immunoassays are the most common types of assays used for ADM and IFX TDM in the clinical space [27].

Although the free drug and free ADA levels would have the most impact on treatment outcome compared to the drug-ADA complexes, the combined quantification of drug and total (free and drug-bound) ADAs still provides clinicians with a more encompassing view of the extent of ADA development in response to the treatment [28,29]. However, the quantification of total ADAs often requires additional processing steps of patient specimens, usually, an acid treatment to dissociate ADA-drug complexes. Consequently, the assay workflow can constitute a time-consuming, multi-step process [30,31,32,33]. Fully automated platforms that feature a simplified procedure with minimal patient sample handling and liquid transfers would benefit operational efficiency and possibly enhance analytical performance.

**Figure 1:**
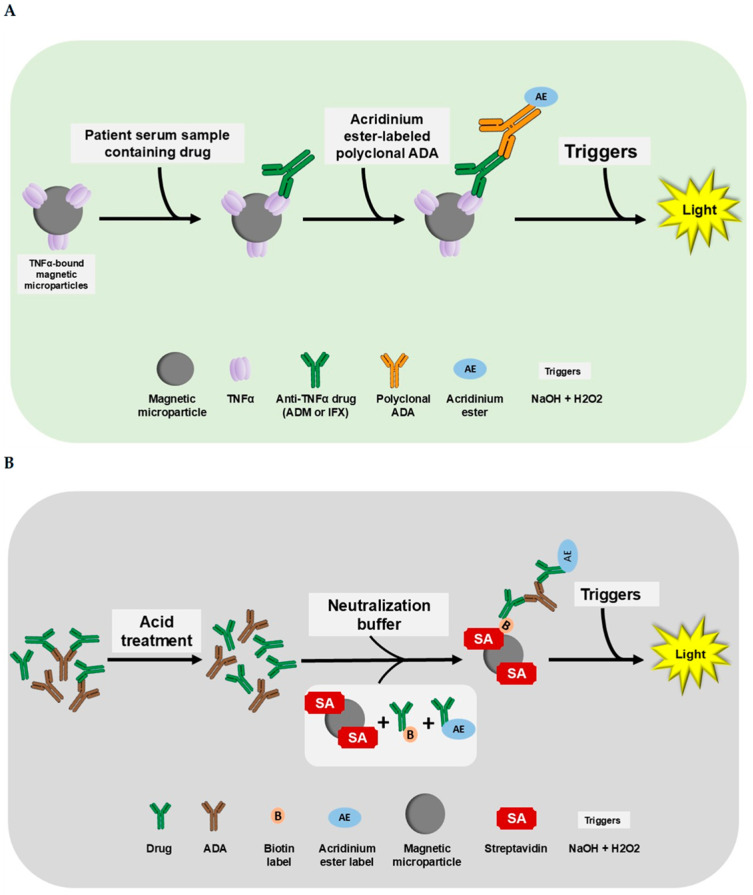
The i-Tracker assay procedure for drug (ADM and IFX) and total ADAs. The addition of reagents and patient serum occurs in cuvettes loaded in the IDS-iSYS analyzer. (**A**) The i-Tracker ADM and IFX drug assay procedure. In the drug assays, the drug in patient serum binds to the TNFα on the magnetic microparticles. After a series of incubation and washing, the final complex formed is exposed to triggers (sodium hydroxide and peroxide). Emission of light proportional to the quantity of Adalimumab in the serum ensues. (**B**) The i-Tracker total ADA procedure. For the quantification of total ADAs, the patient serum first undergoes an acid treatment to dissociate drug and anti-drug complexes. Then, a neutralization buffer containing biotinylated drug and acridinium ester-conjugated drug is added along with streptavidin-bound magnetic microparticles. The final complex formed with the successive addition of reagents is exposed to triggers for light emission. The relative light units (RLUs) obtained on the analyzer are proportional to the total ADAs captured. Illustration created with Microsoft PowerPoint version 2016 (16.0.4266.1001) and NIH BioART source [41].

Along with other emerging automation-based assays, the i-Tracker TDM assays from Biosynex-Theradiag can facilitate the monitoring of ADM, IFX, and associated ADAs. i-Tracker constitutes a family of assays used for the TDM of biotherapies such as ADM and IFX. These assays are provided in a cartridge format and can be run on either of two random-access, multi-disciplinary automated systems: the i-Track10 and the IDS-iSYS analyzers. The i-Tracker assays use a magnetic microparticle-based CLIA technology with acridinium ester as a source of chemiluminescent signal (Figure 1). Various ELISA methodologies (apDia, Mab Track, LISA-Tracker, Sanquin) have been compared to the i-Tracker drug and to both free [34,35] and total ADA assays [36] on i-Track10 and IDS-iSYS by clinical laboratories. However, a more comprehensive validation of the i-Tracker drug and total ADA panels, with key performance factors such as drug tolerance, has not been reported yet. In the present study, we aimed to establish performance characteristics for the i-Tracker ADM, IFX, and associated total ADA assays on the IDS-iSYS analyzer. Our report includes validation parameters [37] such as linearity, imprecision, functional sensitivity, accuracy, and drug tolerance. Electrochemiluminescent immunoassay (ECLIA)-based methods have been developed for drug quantitation. ECLIA-based assays have also been developed as drug-tolerant assays for total ADA quantitation and are adopted in clinical testing [33,38,39,40]. Hence, a method comparison was also carried out between the i-Tracker assay and an ECLIA-based method in this independent validation study.

## 2. Materials and Methods

### 2.1. Materials

The IDS-iSYS analyzer; consumables; and the Biosynex-Theradiag kits, including the i-Tracker Adalimumab Research Use Only (RUO) kit, i-Tracker anti-Adalimumab Total Antibody (Ab) RUO kit, i-Tracker Infliximab RUO kit, i-Tracker anti-Infliximab Total Ab RUO kit, and the external control materials for each of the four assays, were purchased from EUROIMMUN US (Mountain Lakes, NJ, USA). The reference numbers associated with the kits/control material acquired are listed in Table S1. The ADM biosimilar (Bio X Cell Cat# SIM0001, RRID:AB_2894722) and IFX biosimilar (Bio X Cell Cat# SIM0006, RRID:AB_2894727) were acquired from Bio X Cell (Lebanon, NH, USA). The saline for dilutions was acquired from Ricca Chemical Company (Arlington, TX, USA). The monoclonal anti-IFX IgG1 Ab (product code HCA233, clone AbD20436_hIgG1) was purchased from BIO-RAD (Hercules, CA, USA). The polyclonal anti-ADM Ab was provided by Biosynex-Theradiag (Croissy-Beaubourg, France).

### 2.2. Methods

#### 2.2.1. Clinical Specimen Acquisition and Selection

Anonymized remnant serum specimens of 72 ADM- and 99 IFX-treated patients tested in the context of TDM were acquired from a national clinical laboratory. The specimens were sourced based on the clinical detection status of drugs and ADAs, ensuring inclusion of both negative and positive specimens for each individual analyte in the study. Human serum specimens serving as reference materials were randomly selected from a pool of remnant serum samples from routine clinical testing at the Sonic Reference Laboratory. All remnant serum specimens were de-identified prior to placement in the randomly selected pool. As this study was non-interventional and conducted for quality assurance purposes, it was deemed exempt from formal institutional review board (IRB) review. Ethical approval was not required per the Code of Federal Regulations (45 CFR 46.104(d)(4)). The study was conducted in accordance with the Declaration of Helsinki revised in 2024.

#### 2.2.2. Linearity

For the drugs, a pool of randomly selected serum specimens, negative for drugs and ADAs, was spiked either with ADM or with IFX biosimilar to obtain an initial stock solution. The stock solution was 2-fold serially diluted with saline to generate at least five different final drug concentrations. For the ADAs, the manufacturer-provided kit calibrator with the highest concentration was used as the starting material for the serial dilution with saline as a diluent. The linearity of the concentrations obtained from IDS-iSYS was analyzed using a 15% allowable total error (TEa), including a 50% budget for systematic error.

#### 2.2.3. Imprecision

Both intra-assay precision and inter-assay precision were assessed for the i-Tracker assays using a low and high analyte concentration sample. For the intra-assay precision evaluation, each sample was assayed in at least 20 replicates within a single run. For inter-assay precision, each sample was assayed twice daily for 10 days. The data was analyzed with a 15% TEa, including a 50% budget for systematic error.

#### 2.2.4. Functional Sensitivity

The highest calibrator provided for each of the four i-Tracker assays was diluted with saline to obtain 12 different target concentrations to achieve close to the manufacturer-claimed lower limit of quantitation (LLoQ) for each assay. These diluted samples were measured across 10 separate runs. For concentrations below the claimed LLoQ, the assay on the IDS-iSYS analyzer, a best polynomial fit served in the determination of the analyte concentration based on the RLU reported by the IDS-iSYS analyzer. The largest acceptable percent coefficient of variability (% CV) cutoff was set to 20%.

#### 2.2.5. Accuracy for i-Tracker Drug Assays

Accuracy of the drug assays was performed by individually spiking human serum (drug and ADA-free) with a drug biosimilar to achieve concentrations spanning the analytical measurement range (AMR) and beyond. The AMR for each assay was established based on preliminary findings on linearity and method comparison, and to match the calibrator with the highest analyte concentration currently provided by the manufacturer. This ensured proper calibration of the assays as intended by the assay manufacturer. A post-dilution feature on the IDS-iSYS analyzer enabled the programming of a 5-fold and 40-fold post-dilution for the i-Tracker ADM and IFX, respectively, to test specimens with expected above-AMR drug concentrations. A percent difference within ±10% was considered an acceptable target range for accuracy [42,43]. The percent difference was estimated as the percent ratio of the calculated difference between the assigned (theoretical) value and the resulting value to the assigned value.

#### 2.2.6. Method Comparison

For each of the four assays, a comparison was carried out between the results obtained from the i-Tracker assays and those from an electrochemiluminescent immunoassay (ECLIA)-based method used for clinical testing at a national reference laboratory as a laboratory-developed test (LDT). This ECLIA-based method is referred to hereafter as “reference method”. This comparison study is referred to as method comparison. A total of 46, 72, 61, and 99 specimens were used for the method comparison of the i-Tracker ADM, total anti-ADM Ab, IFX, and total anti-IFX Ab assays, respectively. A qualitative analysis (ADA detected versus not detected) was performed for i-Tracker ADA assays. In the discrepancy analysis of the qualitative method comparison for ADA detection, three drug trough concentration levels, which were based on findings for inflammatory bowel disease (IBD) [44,45,46], RA [47,48], and psoriasis [49,50], were used in the classification of drug concentrations. They include subtherapeutic (<5 µg/mL for ADM and <3 µg/mL for IFX), optimal (5–8 µg/mL for ADM and 3–7 µg/mL for IFX), and supratherapeutic range (>8 µg/mL for ADM and >7 µg/mL for IFX). The results from a previously performed functional cell-based assay (CBA) served in the discrepancy analysis on fourteen (14) of the anti-IFX Ab discrepant specimens. The method comparison was conducted in a blinded fashion, where the i-Tracker results obtained at the Sonic Reference Laboratory were not shared with the facility performing either the reference method or the functional CBA. The LLoQ for each assay was utilized as a detection cut-off point for the qualitative analyses. The results related to the reference method are located on the *x*-axes of correlation, bias, and agreement plots, while the *y*-axes feature results pertaining to the i-Tracker assays.

#### 2.2.7. Drug Tolerance

Pooled remnant human serum, which tested negative for drug and ADAs, was spiked either with polyclonal anti-ADM Ab or BIO-RAD anti-Infliximab HCA233 to achieve different concentration levels of ADA. Each anti-ADM concentration level was spiked with eight (8) concentration levels of an ADM biosimilar, to achieve ADM concentrations ranging from 0.6 to 100 µg/mL ADA. Each anti-IFX level was spiked with five (5) concentration levels of an IFX biosimilar to achieve IFX concentrations ranging from 1.5 to 24 µg/mL. These mixtures were left to incubate at room temperature for one (1) hour. After incubation, each mixture was assessed on the IDS-iSYS instrument for ADA concentrations. The drug tolerance of the i-Tracker total ADA kit assay was evaluated by studying the inhibition profile of the measured total ADAs in response to added drug. The LLoQ was used as a detection threshold for each i-Tracker total ADA assay in the qualitative assessment of results. The percent loss in ADA quantitation due to the presence of the drug was calculated.

#### 2.2.8. Statistical Analysis

The linearity, LLoQ, precision, accuracy, and method comparison data were analyzed via EP Evaluator^®^ software version 12.4.0.2 purchased from Data Innovations (Colchester, VT, USA). Microsoft^®^ Office Excel^®^ version 2016 (16.0.5513.1000) purchased from Microsoft Corporation (Redmond, WA, USA) and the OriginPro version 2022b software purchased from OriginLab Corp.(Northampton, MA, USA) were used for the ADA detection status summary in the qualitative method comparison and the drug tolerance plots, respectively. The specimens used for method comparison studies are statistically described as mean ± standard deviation. Quantitative assessment was performed using a deeming regression analysis, where the coefficient of correlation (R), ranging from 0 to 1, was reported. An R-value of 0 indicates no correlation, and an R-value of 1 indicates perfect linear correlation [51]. The deeming regression slope was also reported. When the 95% confidence interval (CI) around the slope does not include 1, then there are proportional differences between the two methods [52]. Quantitative bias was calculated as the difference between values obtained from the i-Tracker method and those from the reference method. A qualitative assessment was performed for the total ADA assays using agreement and Cohen’s Kappa. Agreement is defined as the percentage of i-Tracker cases that match the detection status resulting from the reference method. Hence, in the qualitative assessment of ADAs, positive agreement would be the percent cases that match when the reference method is positive (i.e., analyte detected). Negative agreement would be the percent of cases that match when the reference method is negative (i.e., analyte not detected) [53]. Considering the complex biological assays used for total ADA quantification in this study, an 85% or greater overall qualitative agreement was considered a strong percent agreement [54]. The Cohen’s Kappa assessment produces a degree of agreement after taking into consideration the probability that the two methods agree by chance. A Kappa value in the range of 0–0.20, 0.21–0.39, 0.40–0.59, 0.60–0.79, 0.8–0.90, above 0.90, respectively, indicates no (random) agreement, minimal agreement, weak agreement, moderate agreement, strong agreement and excellent/almost perfect agreement [55]. To assess bias in the qualitative method comparison, the McNemar Test for Symmetry, an Exact test for nominal data, was used. A *p*-value < 0.05 would indicate that a method is consistently larger than the other, and the symmetry test fails [56].

## 3. Results

### 3.1. The i-Tracker Drug and Total ADA Assays Show Linearity and an Imprecision of Less than 8% CV on the IDS-iSYS Analyzer

**Figure 2:**
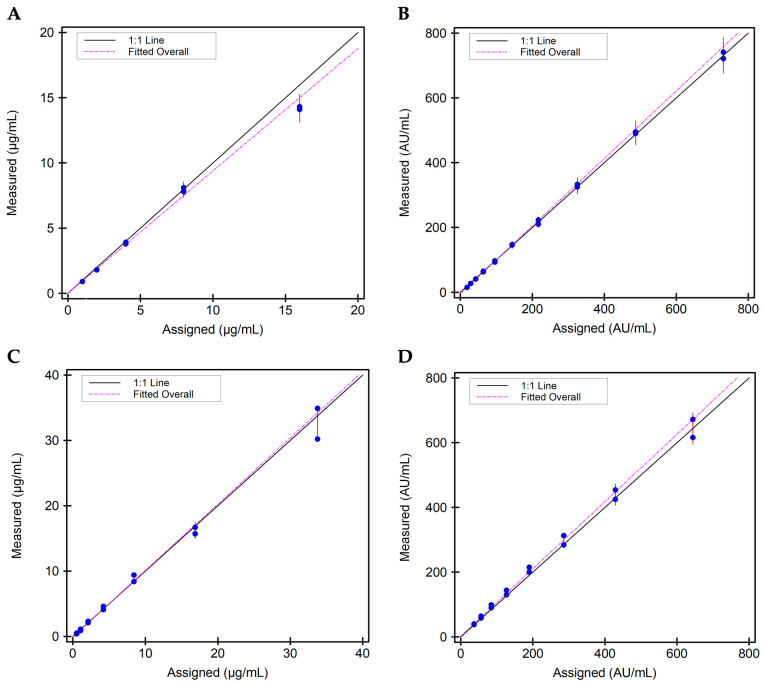
Scatter plots on linearity for the four i-Tracker assays on IDS-iSYS: (**A**) Linearity plot for ADM. Specimens with mean concentrations spanning 0.9 to 14.2 µg/mL were used. (**B**) Linearity plot for total anti-ADM Ab. Specimens with mean concentrations spanning 15.0 to 731.0 AU/mL were used. (**C**) Linearity plot for IFX. Specimens with mean concentrations spanning 0.45 to 32.6 µg/mL were used. (**D**) Linearity plot for total anti-IFX Ab. Specimens with mean concentrations spanning 39.0 to 644.0 AU/mL were used.

In order to assess the proportionality of results obtained via the i-Tracker drug and total ADA assays on IDS-iSYS to the analyte concentration in serum specimens, a linearity study was performed. For ADM (Figure 2A), total anti-ADM Ab (Figure 2B), IFX (Figure 2C), and total anti-IFX (Figure 2D), the results were deemed linear within a TEa of 15%. The estimated slopes were, respectively, 1.088 (6.9% error), 1.043 (3.7% error), 1.015 (5.4% error), and 1.040 (4.3% error) for ADM, IFX, anti-ADM Ab, and anti-IFX Ab. In terms of intra-assay (Table 1) and inter-assay precision (Table 2), all four assays showed less than 8% CV of imprecision for low analyte and high analyte concentration levels. In addition, the observed standard deviation (SD) for each of the four assays was below the 15% TEa-defined SD goal for the analyte concentration level. Collectively, these results demonstrate linearity and an acceptable precision profile of the four i-Tracker assays on the IDS-iSYS analyzer.

### 3.2. The LLoQ Results Obtained on the IDS-iSYS Analyzer Align with the Manufacturer-Claimed Cut-Offs for the i-Tracker Drug and Total ADA Assays

To establish analyte quantitation cutoffs, a LLoQ study was performed using samples resulting from calibrator serial dilution for each of the four assays. The estimated LLoQ values for ADM, total anti-ADM Ab, IFX, and total anti-IFX Ab (Table 3) were slightly lower than the LLoQ values claimed by the assay manufacturer. Additionally, the measurements performed on 28 randomly selected serum specimens, remaining from unrelated clinical testing at the Sonic Reference Laboratory, either showed no detection of the analyte or less than the programmed LLoQ on the IDS-iSYS analyzer for each of the individual assays. Taken together, these LLoQ results establish 0.5 µg/mL and 0.3 µg/mL as quantitative cutoffs for ADM and IFX, respectively. They also confirm 10 AU/mL and 30 AU/mL as appropriate ADA detection cutoffs for the i-Tracker total anti-ADM Ab and total anti-IFX Ab, respectively, on the IDS-iSYS analyzer.

### 3.3. The i-Tracker Drug Assays Show Accuracy Within Their Respective AMR and Beyond on the IDS-iSYS Analyzer

**Figure 3:**
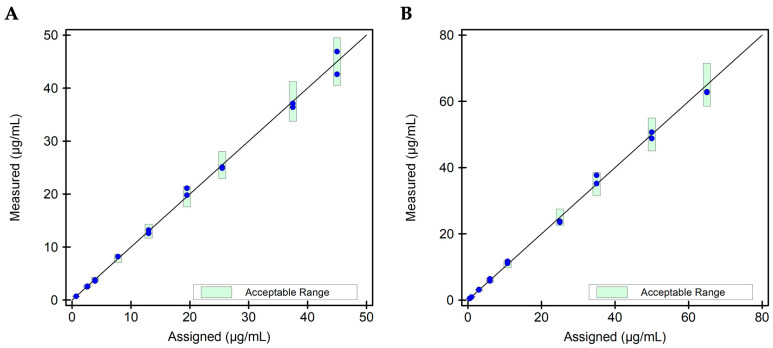
Scatter plot for accuracy evaluation for the i-Tracker drug assays. Two (2) independent replicates of each drug level were tested via the corresponding i-Tracker drug assay on IDS-iSYS. Dilution was enabled in the assay programming to accommodate drug levels above the AMR. (**A**) Scatter plot for the ADM drug assay. Nine (9) specimens with a mean ADM concentration ranging from 0.7 to 45.0 µg/mL were utilized for this study. (**B**) Scatter plot for the IFX drug assay. Nine (9) specimens with a mean IFX concentration ranging from 0.5 to 65.0 µg/mL were utilized for this study. The green bars represent the 10% difference set for the target recovery.

Based on the linearity results described above, the assay specifics for the four analytes were determined as displayed in Table 4. To verify accuracy in measurements via the assays as configured on the IDS-iSYS analyzer, a pool of random remnant serum samples, negative for drug and ADAs, was spiked with a drug biosimilar to obtain drug concentrations in the AMR and beyond. The obtained values on the IDS-iSYS analyzer were then compared to the theoretical concentrations of these spiked samples. Accuracy with a 10% difference range from the expected drug concentration was observed between 0.7 and 45.0 µg/mL ADM (Figure 3A) and between 0.5 and 65.0 µg/mL IFX (Figure 3B). These results confirm accuracy of measurement of drug level via the i-Tracker ADM and IFX assays across their respective AMR and at extended concentrations.

### 3.4. Compared to the Reference Method, i-Tracker Drug Assays Show a Strong Linear Fit Across Their Defined AMR on the IDS-iSYS Analyzer

**Figure 4:**
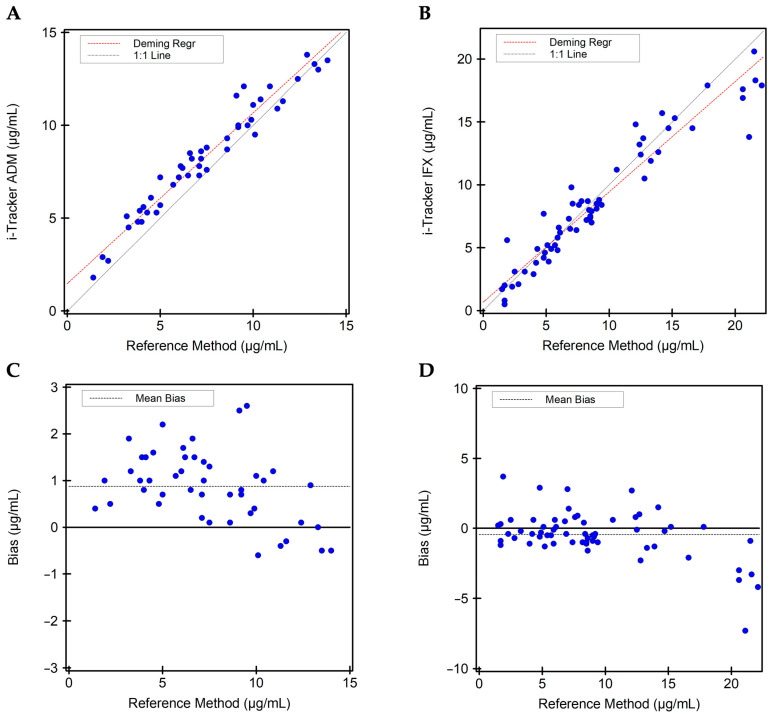
Quantitative method comparison between the i-Tracker drug assays and the reference method. (**A**) Deeming regression analysis for ADM quantitation comparing the i-Tracker ADM assay and reference method. (**B**) Deeming regression analysis for IFX quantitation comparing the i-Tracker IFX assay and reference method. (**C**) Quantitative bias observed for the i-Tracker ADM against the reference method. (**D**) Quantitative bias observed for the i-Tracker IFX against the reference method. In both quantitative bias plots, the *Y*-axis shows the difference between the i-Tracker method and the reference method, with the solid middle line set as 0 µg/mL difference.

A method comparison of the drug assays to an ECLIA-based reference method used in clinical testing was performed (Table 5). For the 46 ADM specimens used, the mean concentrations obtained from the reference method and the i-Tracker ADM assay were, respectively, 7.5 ± 3.3 µg/mL and 8.3 ± 3.0 µg/mL. For the 61 IFX specimens used, the mean concentrations obtained from the reference method and the i-Tracker ADM assay were, respectively, 9.1 ± 5.6 µg/mL and 8.6 ± 5.0 µg/mL. The agreement analysis revealed an R-value of 0.9740 and 0.9568 (deeming regression slope = 0.88), respectively, for ADM (Figure 4A) and IFX (Figure 4B). The deeming regression slope was, respectively, 0.92 (95% CI of 0.86–0.98) and 0.88 (95% CI of 0.81–0.95) for ADM and IFX. The ADM and IFX assays also exhibited, respectively, a mean bias of 0.88 µg/mL (Figure 4C) and −0.44 µg/mL (Figure 4D) compared to the reference method. Together, these results indicate overall strong positive linear correlation with the reference method across the AMR and reveal some level of functional differences in drug quantitation between the i-Tracker method and the reference method.

### 3.5. While Both i-Tracker Total ADA Assays Showed a Strong Postive Agreement with the Reference Method, a Weak Negative Agreement Was Observed for the i-Tracker Total Anti-IFX Ab

**Figure 5:**
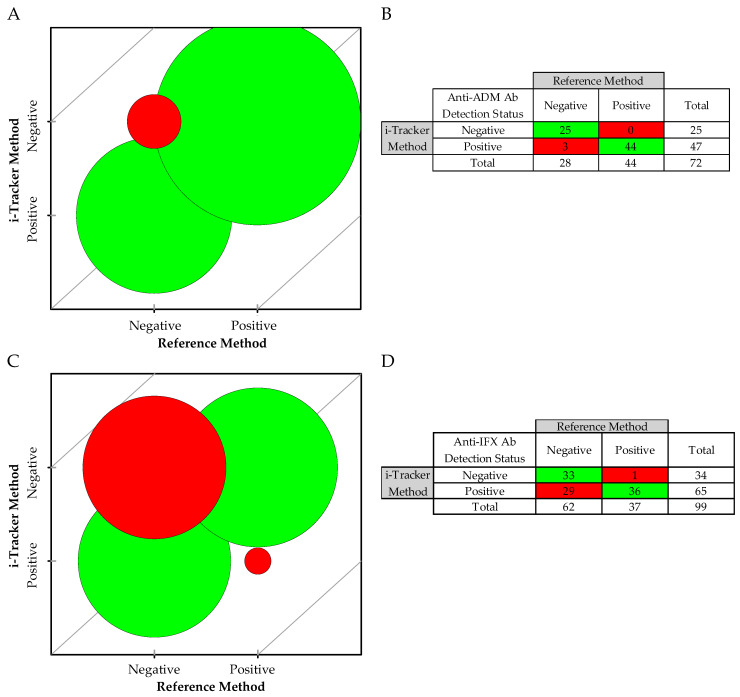
Qualitative method comparison between the i-Tracker total ADA assays and the reference method: (**A**) Bubble agreement chart based on negative (not detected) and positive (detected) for anti-ADM Ab. (**B**) Detection status summary for the 72 remnant specimens from ADM-treated patients. (**C**) Bubble agreement chart based on negative and positive for anti-IFX Ab. (**D**) Detection status summary for the 99 remnant specimens from IFX-treated patients. The red represents mismatches and green represent matching cases between the i-Tracker and the reference method.

Due to non-equivalent ADA concentration units between the two methods, a qualitative assessment was performed for the ADA detection ability of the i-Tracker assays using a “Not Detected” and “Detected” classification. In the method comparison, a total of 72 specimens were used for anti-ADM Ab, and 99 specimens for anti-IFX Ab. A strong agreement was observed for the detection of anti-ADM Ab with a Cohen’s Kappa of 0.91. The McNemar test of symmetry passed (*p* > 0.999), indicating no significant bias between methods. With only three (3) discrepant results, the overall agreement was 95.8%, including 100.0% positive agreement and 89.3% negative agreement (Figure 5A,B). For the anti-IFX Ab, however, a weak agreement was revealed with a Cohen’s Kappa of 0.44, and the McNemar test of symmetry failed (*p* < 0.001). Thirty (30) specimens were discrepant, generating an overall agreement of 69.7% agreement (Figure 5C,D). Interestingly, the qualitative analysis revealed a 97.3% positive agreement and a 53.2% negative agreement, where the i-Tracker assay displayed a higher rate of detection of anti-IFX Ab than the reference method did.

A discrepancy analysis was conducted on the specimens with mismatched classification. In the case of anti-ADM Ab detection, the 3 discrepant specimens showed relatively low total anti-ADM Ab levels as measured via the i-Tracker method, but exhibited drug levels at least in the therapeutic range via both methods (Table 6). For the anti-IFX Ab detection status, additional available TDM data for 14 of the 30 discrepant specimens enabled a more informed discrepancy analysis. Among these 14 specimens, 11 (Specimens 1–11) were also known to be positive by a previously available functional CBA method, designed for detecting drug-neutralizing ADAs (Table 7). In terms of drug level, all three assay types (i.e., i-Tracker assay, the reference method, and the functional CBA) showed concordant results in these 11 specimens, where the drug was either undetected or present at subtherapeutic levels. The remaining three specimens with available results (Specimens 12–14) were found to be negative via the functional CBA method. At least therapeutic levels of the drug were detected in these three specimens by the three assay types. For specimens with no available functional CBA data, which were found positive by the i-Tracker method but negative by the reference method for anti-IFX Ab, the drug was present at either therapeutic or supratherapeutic levels (except Specimens 19 and 28). Only two of these discrepant specimens (Specimens 3 and 5) had sufficient volume remaining for subsequent testing with a third-party ELISA, generally used in clinical testing for the quantification of total ADAs. Both specimens were also found to be positive for the anti-IFX antibodies. Finally, Specimen 30 with a supratherapeutic drug concentration was negative by the i-Tracker method but tested positive at a noted low level for ADA by the reference method.

Taken together, these results indicate comparable qualitative capabilities for the i-Tracker total ADM Ab assay and the reference method in ADA detection. However, the higher ADA detection rate observed with the i-Tracker total anti-IFX Ab assay compared with the reference method underscores functional differences in ADA detection in both methods.

### 3.6. The Quantitation of Total ADAs via the i-Tracker Assays on IDS-iSYS Shows Drug Sensitivity, but There Is No Loss of ADA Detection, Even in Presence of Supratherapeutic Drug Concentrations

**Figure 6:**
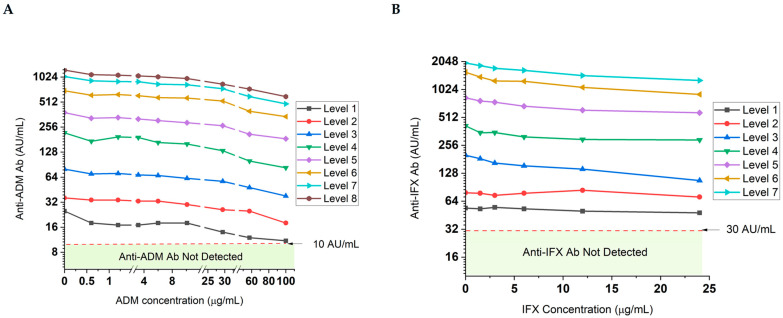
Drug tolerance profile of the total ADA quantitation in serum via the i-Tracker assays on IDS-iSYS. Drug and ADA-negative serum specimens were spiked with different levels of the ADAs and increasing drug concentrations. The levels represent increasing ADA baseline concentrations in serum (before addition of drug), where Level 1 corresponds to the lowest ADA concentration achieved. A log2 scale was used on the *Y*-axis. (**A**) Impact of increasing ADM concentrations on total anti-ADM Ab quantity. Level 1 through 8 are, respectively, 25, 36, 80, 218, 382, 698, 1035, and 1238 AU/mL. Different scales were used in between the line breaks of the *X*-axis to facilitate trend visualization across the drug concentrations used. (**B**) Impact of increasing IFX concentrations on total anti-IFX Ab quantitation. Level 1 through 7 correspond to 54, 79, 200, 415, 831, 1570, and 1973 AU/mL, respectively.

The quantitation of total ADAs is sensitive to the presence of endogenous drugs in serum samples. The i-Tracker total ADA assays feature an initial acid treatment of serum specimens to dissociate drug-ADA complexes prior to addition of the capture antibody and mitigate this interference [36]. To investigate the degree of drug interference in the ADA quantitation by the i-Tracker total anti-ADM Ab and total anti-IFX Ab assays, various drug concentration levels were used to spike different levels of ADA-containing serum samples. A drop in ADA quantity was observed with the first drug level used in the study. For the total anti-ADM Ab assay, 0.6 µg/mL ADM produced a drop of 7 AU/mL (28% loss) in quantity from the initial 25 AU/mL total ADA measured (Figure 6A). In the case of the anti-IFX Ab assay, however, higher drug concentrations (3.0 µg/mL IFX) were needed to generate a greater than 10% drop in total ADA quantitation (Figure 6B). Overall, up to a 62% and 61% decrease in measured ADA AU/mL was observed in the presence of drug concentrations, respectively, for the total anti-ADM Ab and total anti-IFX assay. This impact was generated by 100 µg/mL ADM and 24 µg/mL IFX. The ADAs remained detectable regardless of initial ADA concentration, even in the presence of the highest drug concentrations used in this study. While these results suggest drug sensitivity of the i-Tracker ADA assays in total ADA quantitation, they also highlight the robustness of the assays in detecting the presence of ADAs in serum specimens containing drug concentrations well above clinically relevant drug ranges.

## 4. Discussion

The tandem monitoring of drug and ADA levels offers clinicians a gateway into optimizing the pharmacokinetics of biologics in patients. An accurate view of drug levels and progression of ADA levels can aid in maintaining therapeutic concentrations to sustain clinical benefit and remission in ADM- or IFX-treated patients [44,57,58]. TDM assay platforms with robust analytical performance are, therefore, paramount to this disease management process. In this validation study, we established performance characteristics of the i-Tracker ADM, IFX, and associated total ADA assays on IDS-iSYS, and performed a method comparison of these assays to an ECLIA-based reference method using patient specimens. As a fully automated workflow, running the i-Tracker assays can improve processing time over manual or semi-manual methodologies, albeit with some disadvantages of its own to be considered in laboratory implementation (Table 8). In terms of analytical performance, results from LLOQ studies support the manufacturer’s claimed LLOQ for the i-Tracker drug and ADA assays. Additionally, the four assays displayed an acceptable level of imprecision (<15% CV) for this assay category in clinical testing [59,60,61].

The linearity and accuracy findings for the i-Tracker drug assays demonstrate that the assays produce results proportional and close to the actual analyte concentration in serum specimens on IDS-iSYS. The linearity and precision results align with previous reports concluding satisfactory linearity and precision of i-Tracker drug assays [34,35]. Automated onboard dilution on IDS-iSYS enables the quantification of greater-than AMR drug concentration. As our accuracy results indicate, the reportable range for ADM and IFX levels in patient serum specimens via the i-Tracker drug assays on IDS-iSYS can extend beyond the AMR. The ADA concentrations of commercially available recombinant ADAs are estimated in mass/mL, and quantitative equivalence is not established with the AU/mL obtained via the i-Tracker total ADA assays. Hence, the accuracy in total ADAs quantification could not be assessed. However, the linearity observed for both the total anti-ADM and anti-IFX Ab suggests a strong proportionality between ADA levels present in a sample and the results obtained via the i-Tracker total ADA assays.

Further accuracy studies were performed by method comparison to an ECLIA-based reference method. The choice of this reference method was based on several factors. First, immunoassays are currently mainstream methodologies used by clinical laboratories for the monitoring of ADM and IFX in patient serum [64,65], and therefore, can serve as a reference method in characterizing emerging assays such as the i-Tracker assay. Moreover, different types of ELISAs have already featured in method comparisons against the i-Tracker assays [34,35,36]. Hence, a method comparison with an ECLIA-based method further clarifies the characteristics and limitations of these i-Tracker assays in the TDM of ADM and IFX. Second, both the i-Tracker assays and the reference method are CLIA-type assays, promoting a method comparison on a similar detection chemistry ground.

A quantitative comparison of drug levels showed a strong linear fit and positive correlation between the i-Tracker ADM and IFX assays and the reference method. A minimal quantitative bias was observed. However, the clinical status of patients, whose remnant serum specimens were tested in the method comparison, was not collected. Therefore, the clinical significance of the quantitative bias in the scope of this study is uncertain. Other investigators have also reported a strong positive correlation of the i-Tracker drug assays with ELISA-based methodologies, along with some level of bias [34,35,36]. Multiple factors could be contributing to quantitative bias. These factors include functional differences, analyte stability, sample processing differences between facilities, and differing sensitivities to biological interferants. As common assay interferents, hemolysis, lipemia, and icterus in serum did not cause significant interference on drug quantitation via the i-Tracker drug assays. Furthermore, TNFα, which is part of the i-Tracker drug assay reagent and is present in serum, did not interfere with drug quantitation, up to the supraphysiological concentration of 1 ng/mL. Additionally, the presence of total ADAs up to 1238 AU/mL for ADM and 1973 AU/mL for IFX did not significantly affect drug quantitation. These extraneous findings also demonstrate some level of analytical selectivity for the i-Tracker drug assays. As a full performance characteristics of the reference method for drug quantitation has not been reported yet, the source of bias remains unclear.

In the qualitative comparison conducted for ADA assays, these functional differences were apparent. While an excellent agreement was observed for anti-ADM Ab detection, the anti-IFX Ab assay demonstrates weaker agreement with 30 discrepant samples between the i-Tracker assay and the reference method. Since serum specimens were de-identified and not associated with any clinical characteristics or the specific autoimmune disease of the patient, the anti-IFX Ab discrepant sample analysis was limited to analytical investigation. For a subset of remnant samples, ADA detection results, obtained via functional CBA used for the detection of drug-neutralizing ADAs, were available. These results allowed for resolution of 11 discrepant anti-IFX results in favor of the i-Tracker assay. Three samples tested negative by both the functional CBA method and by the reference method but tested positive for anti-IFX Ab by the i-Tracker assay. Interestingly, all three of these specimens contained IFX at therapeutic or supratherapeutic levels; so did 13 additional specimens noted for discrepant results between the i-Tracker and the reference methods.

While it is plausible that these discrepant specimens may represent false positives for the i-Tracker total anti-IFX Ab assay, the results from LLoQ studies and remnant human serum serving as a reference material contradict this notion. Additionally, more than a third of discrepant specimens had no detectable drug or subtherapeutic levels of the drug, which would be consistent with presence of ADA. These observations indicate false positives are not likely to be a major contributor to the discrepant results. A third-party ELISA designed for the quantification of total anti-IFX Ab was available for additional analysis. However, low sample volume restricted additional testing to only two specimens. The results from the ELISA method corroborated the presence of anti-IFX Ab as reported by the i-Tracker assay. As a drug target, presence of TNFα could potentially act as an interferant since the labeled drug is used both as a capture and detection antibody in the i-Tracker ADA assays. Spiking experiments with recombinant TNFα at concentrations up to 1.0 ng/mL in pooled remnant serum, negative for drug and ADAs, did not generate false positive ADA results. Moreover, increasing levels of TNFα in anti-IFX Ab patient samples did not significantly interfere with total anti-IFX Ab quantitation on IDS-iSYS. Taken together, these observations suggest that the detection status resulting from the i-Tracker total anti-IFX Ab in the study was not a false positive. Although performed with a low number of specimens, the discrepancy analysis involving the functional CBA reveals that the i-Tracker total anti-IFX Ab assay can detect both drug-neutralizing and non-drug-neutralizing ADAs.

The discrepancies observed in the method comparison, especially the poor agreement between the i-Tracker total anti-IFX Ab assay and its ECLIA-reference method counterpart, highlight functional differences between the i-Tracker CLIA method and the reference method. The discrepancy analysis conducted with some of the specimens suggests differing antigenic and affinity properties of antibodies between the two methods and potentially a difference in sensitivity for the presence of the drug when detecting anti-IFX Ab. Indeed, a similar observation of the higher ADA detection rate by the i-Tracker total anti-IFX Ab assay has also been reported in a method comparison with the ELISA-based Sanquin method, in presence of therapeutic drug levels [36]. These observations point to a potentially higher threshold for drug sensitivity of the i-Tracker assay compared to the reference method. The drug tolerance profile of the i-Tracker total ADA assays indicates some degree of drug sensitivity, but also no loss of anti-IFX Ab detection in presence of supratherapeutic drug concentrations. Differences in levels of drug sensitivity between the i-Tracker method and the reference method could, therefore, affect ADA detectability when specimens contain drug-ADA complexes. In alignment with results from the drug tolerance studies, we observed that the presence of the drug, as displayed in the method comparison discrepancy analysis tables above, did not hinder ADA detection via the i-Tracker total ADA assay for all, except one specimen used in this study. As the status of drug sensitivity of the anti-IFX Ab assay via the reference method is not known, its impact on the ADA detection discrepancies observed remains to be confirmed. Together, the performance characteristics reported in the present validation study may facilitate a more informed interpretation of clinical results when generated by the i-Tracker drug and total ADA assays.

## 5. Clinical Implications

The heterogeneity of ADAs within an individual and inter-individual variability can affect affinity and avidity characteristics in the various ADA assays available [66,67]. Additionally, analyte sensitivity, cross-reactivity, matrix effects, or interference may differ between assays. Hence, discrepancies are not uncommon in method comparisons, especially for ADA detection and quantitation, which have no established international analyte standards [68,69,70]. With each assay having its own advantages and limitations, it becomes critical to clarify the type of result output for each assay. Beyond these potential assay differences, the high rate of ADA positivity and the subsequent discrepancies in our method comparison also highlight the complexity that supersensitive ADA assays add to interpreting TDM results [71]. The significance of ADAs in the presence of therapeutic and supratherapeutic drug levels is not completely understood. Various studies show the ADA incidence rate in inflammatory bowel disease exceeding 40% [72,73]. Specifically for IFX, a long-term efficacy study in Crohn’s disease reported that 61% of patients had detectable ADA after the fifth infusion, but only 37% of patients had a high titer of ADA [74]. A growing number of studies indicate that clinically relevant ADA, those associated with increased risk of infusion reactions and loss of response, are associated with persistent ADA with moderate to high titers [75,76].

In a clinical setting, patients who have serum drug levels within at least the therapeutic range but with detectable ADA would be followed with additional testing and closely monitored for progression or regression of symptoms as recommended by the American Gastroenterological Association Institute and others [16,75,77]. ADAs in these cases may be transient and non-neutralizing. However, when ADA is persistent, at higher levels, and associated with undetectable or subtherapeutic trough concentrations, a neutralizing antibody is suspected. In these scenarios, switching to an alternative biologic therapy may be more effective [16]. Robust clinical studies are largely lacking for commercially available ADA assays to establish optimal ADA cutoffs for high and low level antibodies. In light of these needs, our findings indicate that the i-Tracker ADA assays could have some utility in both proactive and reactive TDM. Like other assays featuring a relatively high drug tolerance, the i-Tracker total ADA assays could potentially support the early detection of ADA during treatment, even in presence of supratherapeutic drug concentrations. However, disease activity and treatment response would still be primary endpoints in determining the need for therapeutic intervention in patients. Considering the methodological differences between the various TDM methods often reported by similar validation studies, and as our study has also shown, it is critical that their detection capabilities and limitations are clarified. Moreover, the use of the same methodology format—if validated for this purpose—would be recommended to ensure TDM continuity in patients.

## 6. Study Limitations

While we established performance characteristics of the i-Tracker drug and total ADA assays on IDS-iSYS, there are limitations to some aspects of this study. First, in addition to the lack of clinical characteristics associated with the patient specimens used in the method comparison, both the reference method and functional CBA were conducted at a different facility. These study conditions limit our ability to evaluate potential sources of discrepancy beyond those already discussed above. To minimize methodological impact, a reference laboratory, where the reference method has been thoroughly validated for clinical testing, was selected for the method comparison. Second, the measurements in the method comparison were only performed once, due to limited sample volume. However, internal and external controls were included in our procedure to ensure quality of the results. Third, the results obtained in the method comparison only pertain to the reference method selected, and may differ based on the performance and functional characteristics of other methods. Fourth, a drug biosimilar was used to spike serum in the accuracy and linearity studies of the drug assays, and a commercial anti-drug Ab was used for drug tolerance studies of ADA assays. This was performed to achieve drug concentrations within the AMR and above the AMR for these studies. The strong linear fit observed with the quantitative method comparison using ADM and IFX patient specimens still demonstrates that i-Tracker assays can produce results that correlate with the drug levels in patient samples. In addition, the strong analytical performance observed with the biosimilar indicates the suitability of the i-Tracker drug assays in the monitoring of patients treated with ADM or IFX biosimilar. For the drug tolerance studies, a differential impact of the drug on ADA quantitation has been reported when using patient ADA specimens versus serum spiked with the commercial ADA. In a study evaluating the drug tolerance of the fluoroenzyme immunoassay-based method using the ImmunoCap solid phase and run on the Phadia^TM^ 250 automated platform for the acid-free detection of ADAs, the presence of the drug generated a greater loss of ADA signal on the serum spiked with the commercial anti-IFX Ab than on IFX-treated patient specimens [30]. Considering these reports and our findings with the same commercial ADA in the drug tolerance study, using patient specimens is anticipated to produce a stronger drug tolerance profile for the i-Tracker total ADA assays.

## 7. Conclusions

In this validation study, we provided performance characteristics of the i-Tracker assay panel for ADM, IFX, and associated total ADA quantitation on the IDS-iSYS analyzer. To our knowledge, this is the first report featuring a validation including drug tolerance on these i-Tracker assays by a clinical laboratory in the United States. Our findings showed a robust analytical performance from the i-Tracker ADM, IFX, and associated ADA assays on the IDS-iSYS platform, indicating that the assays meet the criteria for the clinical monitoring of ADM- and IFX-treated patients in a high-throughput environment. Moreover, we provide a first glance at the method comparison of an ECLIA-based reference method to the i-Tracker CLIA assay system. As our findings revealed functional differences between the two types of assays, especially regarding anti-IFX Ab detection, switching methodologies would require considering these differences and limitations of each assay to ensure that the goal of the TDM, whether proactive or reactive, can be supported by the methodology.

## Figures and Tables

**Table 1:** Intra-assay precision characteristics for the four i-Tracker assays on IDS-iSYS.

	Low Analyte Concentration			High Analyte Concentration		
	Mean	SD	%CV	Mean	SD	%CV
ADM	2.83	0.06	2.2	12.13	0.7	5.1
Total Anti-ADM Ab	55.5	1.2	0.7	491.5	5.9	1.2
IFX	4.29	0.19	4.4	13.27	0.76	5.8
Total Anti-IFX Ab	75.2	1.0	1.3	1880.4	47.8	2.5

**Table 2:** Inter-assay precision characteristics for the four i-Tracker assays on IDS-iSYS.

	Low Analyte Concentration			High Analyte Concentration		
	Mean	SD	%CV	Mean	SD	%CV
ADM	3.11	0.14	4.4	13.22	0.91	6.9
Total Anti-ADM Ab	390.9	19.1	4.9	1009.4	28.3	2.8
IFX	2.70	0.2	7.6	17.5	1.08	6.2
Total Anti-IFX Ab	59.6	2.6	4.4	1169.8	65.0	5.6

**Table 3:** LLoQ results for the four i-Tracker assays on IDS-iSYS.

Assays	Calculated LLoQ	Claimed LLoQ
ADM	<0.43 µg/mL	0.5 µg/mL
Total Anti-ADM Ab	7.86 AU/mL	10 AU/mL
IFX	0.28 µg/mL	0.3 µg/mL
Total Anti-IFX Ab	<28 AU/mL	30 AU/mL

**Table 4:** Assay specifics of the i-Tracker ADM, IFX, and associated total ADA monitoring on IDS-iSYS.

	ADM		IFX	
Analytes	Drug	Total ADA	Drug	Total ADA
Units	µg/mL	AU/mL	µg/mL	AU/mL
AMR	0.5–14	10–2000	0.3–24	30–2000

**Table 5:** Assay specifics of the ECLIA-based reference method.

	ADM		IFX	
Analytes	Drug	Total ADA	Drug	Total ADA
Units	µg/mL	ng/mL	µg/mL	ng/mL
Methodology	Meso Scale Discovery ECLIA (plate-based)			
Reportable range	0.4–40	20–1500	0.5–40	20–1500

**Table 6:** Discrepancy analysis of qualitative mismatches between the i-Tracker total anti-ADM Ab assay and the reference method.

Drug Concentration			ADA Detection Status	
Specimen Number	i-Tracker Method (µg/mL)	Reference Method (µg/mL)	i-Tracker Method (AU/mL)	Reference Method (ng/mL)
1	7.3	6.5	Detected (41)	Not Detected
2	13.5	14	Detected (11)	Not Detected
3	5.7	5	Detected (55)	Not Detected

**Table 7:** Discrepancy analysis of qualitative mismatches between the i-Tracker total anti-IFX Ab assay and the reference method.

Drug Concentration				ADA Detection Status		
Specimen Number	i-Tracker Method (µg/mL)	Reference Method (µg/mL)	Functional CBA(µg/mL)	i-Tracker Method (AU/mL)	Reference Method (ng/mL)	Functional CBA
1	<0.3	<0.5	Not Detected	Detected (332)	Not Detected	Detected
2	<0.3	<0.5	Not Detected	Detected (440)	Not Detected	Detected
3	<0.3	<0.5	Not Detected	Detected (472)	Not Detected	Detected
4	<0.3	<0.5	Not Detected	Detected (280)	Not Detected	Detected
5	<0.3	<0.5	Not Detected	Detected (476)	Not Detected	Detected
6	<0.3	<0.5	Not Detected	Detected (546)	Not Detected	Detected
7	<0.3	<0.5	Not Detected	Detected (186)	Not Detected	Detected
8	<0.3	<0.5	Not Detected	Detected (165)	Not Detected	Detected
9	0.5	1.7	Not Detected	Detected (724)	Not Detected	Detected
10	<0.3	<0.5	Not Detected	Detected (474)	Not Detected	Detected
11	<0.3	<0.5	Not Detected	Detected (113)	Not Detected	Detected
12	13.2	12.4	16.49	Detected (252)	Not Detected	Not Detected
13	12.4	12.5	16.06	Detected (787)	Not Detected	Not Detected
14	4.8	5.9	6.33	Detected (110)	Not Detected	Not Detected
15	7.0	8.6	Not available	Detected (528)	Not Detected	Not available
16	8.5	7.1		Detected (75)	Not Detected	
17	5.8	5.9		Detected (163)	Not Detected	
18	9.8	7		Detected (586)	Not Detected	
19	2.9	4.0		Detected (37)	Not Detected	
20	13.8	21.1		Detected (455)	Not Detected	
21	10.5	12.8		Detected (1204)	Not Detected	
22	15.3	15.2		Detected (44)	Not Detected	
23	4.9	5.4		Detected (206)	Not Detected	
24	20.6	21.5		Detected (41)	Not Detected	
25	13.7	12.7		Detected (271)	Not Detected	
26	8.4	7.6		Detected (147)	Not Detected	
27	3.9	5.2		Detected (160)	Not Detected	
28	<0.3	<0.5		Detected (146)	Not Detected	
29	6.2	6.1		Detected (47)	Not Detected	
30	7.9	8.6		Not Detected	Detected (51)	

**Table 8:** Procedural considerations for the i-Tracker Adalimumab and Infliximab TDM on IDS-iSYS compared to manual or semi-automated assays.

Methodologies for ADM and IFX TDM Panel	Advantages	Disadvantages	References
Manual or semi-automated TDM assays	Large body of literature on assay performance and on method comparisons, providing references for laboratory validations	Risk of errors in sample processing or in data input	[33,34,62,63]
	Often used in clinical testing, which supports reliability	Lengthier procedure (about 2 h or more)	
	More control over assay development process	Several liquid transfers, which can result in loss of analyte	
	More control over consumables and reagent use	Risk of technologist fatigue, depending on the extent of process automation	
	Potentially cost-effective		
i-Tracker TDM assays on IDS-iSYS	Shorter procedure (less than one hour/sample), which can improve turnaround time and assay troubleshooting time	No random access with other non-TDM assays on the instrument *	[34,35,36]
	Automated data transfer into laboratory information management system	Less data on assay performance in the clinical arena	
	Ready-to-use reagents in a cartridge format, including an acid buffer for total ADA quantification	Less control over consumables and reagent use by instrument, leading to potential waste	
	Adjustable AMR configuration with post-dilution features, enabling additional assay development and an extendable reportable range	Potentially more expensive	
	Hands-off sample processing, with potential for standardization	Risk of instrument failure, leading to specimen processing delays	

* this limitation is due to the potential interference from the specific decontaminating solution necessary for the TDM handling process, with other non-immunoassays.

## Data Availability

The original contributions presented in this study are included in the article/supplementary material. Further inquiries can be directed to the corresponding authors.

## Supplementary Materials

The following supporting information can be downloaded at https://www.mdpi.com/article/10.3390/diagnostics15192447/s1. Table S1: Reference numbers of Biosynex-Theradiag kits used in the study.

## Abbreviations

The following abbreviations are used in this manuscript:

Ab	Antibody
ADA	Anti-drug antibody
ADM	Adalimumab
AMR	Analytical measurement range
CBA	Cell-based assay
CLIA	Chemiluminescent immunoassay
ECLIA	Electrochemiluminescent immunoassay
IBD	Inflammatory bowel disease
IFX	Infliximab
IRB	Institutional review board
LDT	Laboratory-developed test
LLoQ	Lower limit of quantitation
RA	Rheumatoid arthritis
RLU	Relative light unit
TDM	Therapeutic drug monitoring

## References

[1] Yasmeen F., Pirzada R.H., Ahmad B., Choi B., Choi S. (2024). Understanding autoimmunity: Mechanisms, predisposing factors, and cytokine therapies. Int. J. Mol. Sci..

[2] Souza R.F., Caetano M.A.F., Magalhaes H.I.R., Castelucci P. (2023). Study of tumor necrosis factor receptor in the inflammatory bowel disease. World J. Gastroenterol..

[3] Garcia-Carbonell R., Yao S.J., Das S., Guma M. (2019). Dysregulation of intestinal epithelial cell ripk pathways promotes chronic inflammation in the ibd gut. Front. Immunol..

[4] Jang D.I., Lee A.H., Shin H.Y., Song H.R., Park J.H., Kang T.B., Lee S.R., Yang S.H. (2021). The role of tumor necrosis factor alpha (tnf-alpha) in autoimmune disease and current tnf-alpha inhibitors in therapeutics. Int. J. Mol. Sci..

[5] Song Y., Li J., Wu Y. (2024). Evolving understanding of autoimmune mechanisms and new therapeutic strategies of autoimmune disorders. Signal Transduct. Target. Ther..

[6] Ellis C.R., Azmat C.E. (2025). Adalimumab. Statpearls.

[7] Mouser J.F., Hyams J.S. (1999). Infliximab: A novel chimeric monoclonal antibody for the treatment of crohn’s disease. Clin. Ther..

[8] Fatima R., Bittar K., Aziz M. (2025). Infliximab. Statpearls.

[9] Mitoma H., Horiuchi T., Tsukamoto H., Ueda N. (2018). Molecular mechanisms of action of anti-tnf-α agents-comparison among therapeutic tnf-α antagonists. Cytokine.

[10] Scallon B.J., Moore M.A., Trinh H., Knight D.M., Ghrayeb J. (1995). Chimeric anti-tnf-alpha monoclonal antibody ca2 binds recombinant transmembrane tnf-alpha and activates immune effector functions. Cytokine.

[11] Vande Casteele N., Ferrante M., Van Assche G., Ballet V., Compernolle G., Van Steen K., Simoens S., Rutgeerts P., Gils A., Vermeire S. (2015). Trough concentrations of infliximab guide dosing for patients with inflammatory bowel disease. Gastroenterology.

[12] Dehoorne J.L., Groth H., Carlé E., De Schrijver I., Sys C., Delbeke P., Kreps E.O., Renson T., Bonroy C. (2023). Defining a therapeutic range for adalimumab serum concentrations in the management of pediatric noninfectious uveitis, a step towards personalized treatment. Pediatr. Rheumatol. Online J..

[13] Owczarczyk-Saczonek A., Owczarek W., Osmola-Mańkowska A., Adamski Z., Placek W., Rakowska A. (2019). Secondary failure of tnf-α inhibitors in clinical practice. Dermatol. Ther..

[14] Rivera R., Herranz P., Vanaclocha F. (2014). Clinical significance of immunogenicity in biologic therapy. Actas Dermosifiliogr..

[15] Strand V., Goncalves J., Isaacs J.D. (2021). Immunogenicity of biologic agents in rheumatology. Nat. Rev. Rheumatol..

[16] Feuerstein J.D., Nguyen G.C., Kupfer S.S., Falck-Ytter Y., Singh S. (2017). American gastroenterological association institute guideline on therapeutic drug monitoring in inflammatory bowel disease. Gastroenterology.

[17] Alavi A., Loftus E.V. (2023). Adalimumab therapeutic drug monitoring improves treatment outcome in patients with psoriasis. J. Investig. Dermatol..

[18] Mitchell R.A., Shuster C., Shahidi N., Galorport C., DeMarco M.L., Rosenfeld G., Enns R.A., Bressler B. (2016). The utility of infliximab therapeutic drug monitoring among patients with inflammatory bowel disease and concerns for loss of response: A retrospective analysis of a real-world experience. Can. J. Gastroenterol. Hepatol..

[19] Papamichael K., Cheifetz A.S., Melmed G.Y., Irving P.M., Casteele N.V., Kozuch P.L., Raffals L.E., Baidoo L., Bressler B., Devlin S.M. (2019). Appropriate therapeutic drug monitoring of biologic agents for patients with inflammatory bowel diseases. Clin. Gastroenterol. Hepatol..

[20] Suh K., Kyei I., Hage D.S. (2022). Approaches for the detection and analysis of antidrug antibodies to biopharmaceuticals: A review. J. Sep. Sci..

[21] Smeijsters E.H., van der Elst K.C.M., Visch A., Gobel C., Loeff F.C., Rispens T., Huitema A.D.R., van Luin M., El Amrani M. (2023). Optimization of a quantitative anti-drug antibodies against infliximab assay with the liquid chromatography-tandem mass spectrometry: A method validation study and future perspectives. Pharmaceutics.

[22] Gaspar V.P., Ibrahim S., Zahedi R.P., Borchers C.H. (2021). Utility, promise, and limitations of liquid chromatography-mass spectrometry-based therapeutic drug monitoring in precision medicine. J. Mass Spectrom..

[23] Tron C., Lemaitre F., Bros P., Goulvestre C., Franck B., Mouton N., Bagnos S., Coriat R., Khoudour N., Lebert D. (2022). Quantification of infliximab and adalimumab in human plasma by a liquid chromatography tandem mass spectrometry kit and comparison with two elisa methods. Bioanalysis.

[24] Jourdil J.-F., Némoz B., Gautier-Veyret E., Romero C., Stanke-Labesque F. (2018). Simultaneous quantification of adalimumab and infliximab in human plasma by liquid chromatography–tandem mass spectrometry. Ther. Drug Monit..

[25] Kim E.S., Chon H., Kwon Y., Lee M., Kim M.J., Choe Y.H. (2024). Fluorescence-based lateral flow immunoassay for quantification of infliximab: Analytical and clinical performance evaluation. Ther. Drug Monit..

[26] Bonazzi E., Maniero D., Lorenzon G., Bertin L., Bray K., Bahur B., Barberio B., Zingone F., Savarino E.V. (2024). Comparing point-of-care technology to elisa testing for infliximab and adalimumab levels in adult inflammatory bowel disease patients: A prospective pilot study. Diagnostics.

[27] Gorovits B., Baltrukonis D.J., Bhattacharya I., Birchler M.A., Finco D., Sikkema D., Vincent M.S., Lula S., Marshall L., Hickling T.P. (2018). Immunoassay methods used in clinical studies for the detection of anti-drug antibodies to adalimumab and infliximab. Clin. Exp. Immunol..

[28] Wadhwa M., Cludts I., Atkinson E., Rigsby P. (2025). The first who reference panel for infliximab anti-drug antibodies: A step towards harmonizing therapeutic drug monitoring. Front. Immunol..

[29] Dreesen E., Bossuyt P., Mulleman D., Gils A., Pascual-Salcedo D. (2017). Practical recommendations for the use of therapeutic drug monitoring of biopharmaceuticals in inflammatory diseases. Clin. Pharmacol..

[30] Karsten C., Grannas K., Bergman O., Moverare R., Roforth M., Willrich M.A.V., Snyder M.R., Yang Y.K. (2024). Evaluating the performance of two automated anti-drug antibodies assays for infliximab and adalimumab without acid dissociation. AAPS J..

[31] Francois F., Naimi L., Roblin X., Berger A.E., Paul S. (2021). Adalimumab and anti-adalimumab lisa-tracker immunoassays performance criteria for therapeutic drug monitoring of adalimumab-amgen biosimilar (abp501). BMC Immunol..

[32] Cerutti H., Tesi G., Petrini F., Bandini T., Cartocci A., Ianniello A., Bogi A., Muzzi C., Brogi A. (2024). Detection of infliximab, adalimumab, and anti-drug antibodies: Development and validation of new monotest, automated assays on multiparametric instrument. Pract. Lab. Med..

[33] Profaizer T., Elgort M.G., Delgado J.C. (2025). Development and laboratory validation of an electrochemiluminescence elisa technique for measuring infliximab concentrations and anti-drug antibodies. J. Immunol. Methods.

[34] Berger A.E., Gleizes A., Waeckel L., Roblin X., Krzysiek R., Hacein-Bey-Abina S., Soriano A., Paul S. (2022). Validation study of a new random-access chemiluminescence immunoassay analyzer i-track10((r)) to monitor infliximab and adalimumab serum trough levels and anti-drug antibodies. Int. J. Mol. Sci..

[35] Herroelen P., Vanpoucke H., Baert F., Decavele A.S., De Cuyper I., Debrabandere J., Martens G.A., De Smet D. (2024). Analytical and diagnostic performance of theradiag i-tracker assays on ids-isys for infliximab and adalimumab therapeutic drug monitoring. Clin. Chem. Lab. Med..

[36] Vroemen W.H.M., Agata S.S., van Beers J., Damoiseaux J. (2024). Therapeutic drug monitoring of infliximab and adalimumab through concentration and anti-drug antibodies assessment; comparison of sanquin diagnostics and theradiag assays. Antibodies.

[37] Andreasson U., Perret-Liaudet A., van Waalwijk van Doorn L.J., Blennow K., Chiasserini D., Engelborghs S., Fladby T., Genc S., Kruse N., Kuiperij H.B. (2015). A practical guide to immunoassay method validation. Front. Neurol..

[38] Adalimumab and Anti-Adalimumab Antibody, Doseassure™ Adl. Labcorp. https://www.labcorp.com/tests/503890/adalimumab-and-anti-adalimumab-antibody-doseassure-adl.

[39] Infliximab and Anti-Infliximab Antibody, Doseassure™ Ifx. Labcorp. https://www.labcorp.com/tests/503870/infliximab-and-anti-infliximab-antibody-doseassure-ifx.

[40] (2025). Infliximab Quantitation with Reflex to Antibodies to Infliximab, Serum. Mayo Clinic Laboratories. https://www.mayocliniclabs.com/test-catalog/overview/63437#Performance.

[41] (2024). NIAID Visual & Medical Arts. *Antibody*. NIAID NIH BIOART Source. https://bioart.niaid.nih.gov/bioart/17.

[42] Tiwari G., Tiwari R. (2010). Bioanalytical method validation: An updated review. Pharm. Methods.

[43] US FDA (2018). Bioanalytical Method Validation Guidance for Industry. https://www.fda.gov/media/70858/download.

[44] Juncadella A., Papamichael K., Vaughn B.P., Cheifetz A.S. (2018). Maintenance adalimumab concentrations are associated with biochemical, endoscopic, and histologic remission in inflammatory bowel disease. Dig. Dis. Sci..

[45] Kolho K.L. (2020). Therapeutic drug monitoring and outcome of infliximab therapy in pediatric onset inflammatory bowel disease. Front. Pediatr..

[46] Vande Casteele N., Khanna R., Levesque B.G., Stitt L., Zou G.Y., Singh S., Lockton S., Hauenstein S., Ohrmund L., Greenberg G.R. (2015). The relationship between infliximab concentrations, antibodies to infliximab and disease activity in crohn’s disease. Gut.

[47] Nakae K., Masui S., Yonezawa A., Hashimoto M., Watanabe R., Murata K., Murakami K., Tanaka M., Ito H., Yokoyama K. (2021). Potential application of measuring serum infliximab levels in rheumatoid arthritis management: A retrospective study based on kurama cohort data. PLoS ONE.

[48] Pouw M.F., Krieckaert C.L., Nurmohamed M.T., van der Kleij D., Aarden L., Rispens T., Wolbink G. (2015). Key findings towards optimising adalimumab treatment: The concentration–effect curve. Ann. Rheum. Dis..

[49] Menting S.P., Coussens E., Pouw M.F., van den Reek J.M.P.A., Temmerman L., Boonen H., de Jong E.M.G.J., Spuls P.I., Lambert J. (2015). Developing a therapeutic range of adalimumab serum concentrations in management of psoriasis: A step toward personalized treatment. JAMA Dermatol..

[50] Dannepond C., Maruani A., Machet L., Ternant D., Paintaud G., Samimi M. (2015). Serum infliximab concentrations in psoriatic patients treated with infliximab: A systematic review. Acta Derm. Venereol..

[51] Francq B.G., Govaerts B.B. (2014). Measurement methods comparison with errors-in-variables regressions. From horizontal to vertical ols regression, review and new perspectives. Chemom. Intell. Lab. Syst..

[52] Lee S. (2025). Agreement evaluation in statistical analyses: Misconceptions and key features. Ann. Lab. Med..

[53] Watson P.F., Petrie A. (2010). Method agreement analysis: A review of correct methodology. Theriogenology.

[54] Elliott R.J., Pourmohamad T., Webb-Vargas Y., Yan W., Nijem I., Siguenza P., Song Y. (2024). Bioanalytical method comparison strategy for clinical anti-drug antibody immunoassays. AAPS J..

[55] McHugh M.L. (2012). Interrater reliability: The kappa statistic. Biochem. Med..

[56] Sundjaja J.H., Shrestha R., Krishan K. (2025). Mcnemar and mann-whitney u tests. Statpearls.

[57] Syversen S.W., Jorgensen K.K., Goll G.L., Brun M.K., Sandanger O., Bjorlykke K.H., Sexton J., Olsen I.C., Gehin J.E., Warren D.J. (2021). Effect of therapeutic drug monitoring vs. standard therapy during maintenance infliximab therapy on disease control in patients with immune-mediated inflammatory diseases: A randomized clinical trial. JAMA.

[58] Li Y., Xie C., Ding X., Wu Z., Zhang J., Zhu J., Miao L. (2024). What are the benefits of therapeutic drug monitoring in the optimization of adalimumab therapy? A systematic review and meta-analysis up to 2022. Front. Pharmacol..

[59] Senant M., Musset L., Chyderiotis G., Guis-Cabanne L., Damoiseaux J., Fabien N., Dragon-Durey M.-A. (2020). Precision of autoantibody assays in clinical diagnostic laboratories: What is the reality?. Clin. Biochem..

[60] Wnendt S. (2014). FDA raises the bar in bioanalytical method validation. J. Clin. Stud..

[61] Lebo T., Merkel P.A., Knight V., Schmitz J.L., Detrick B., O’Gorman M.R.G. (2024). Validation and Quality Control: General Principles and Application to the Clinical Immunology Laboratory. Manual of Molecular and Clinical Laboratory Immunology.

[62] Rohner F., Zeder C., Zimmermann M.B., Hurrell R.F. (2005). Comparison of manual and automated elisa methods for serum ferritin analysis. J. Clin. Lab. Anal..

[63] Tacker D.H., Topardo J., Mahaffey C., Perrotta P.L. (2014). Workflow analysis comparing manual and automated specimen processing for mass spectrometry–based vitamin d testing. Lab. Med..

[64] van Bezooijen J.S., Koch B.C.P., van Doorn M.B.A., Prens E.P., van Gelder T., Schreurs M.W.J. (2016). Comparison of three assays to quantify infliximab, adalimumab, and etanercept serum concentrations. Ther. Drug Monit..

[65] Jukic T., Drobne D., Pusavec S., Ihan A., Stubljar D., Starc A. (2023). Comparison of 3 enzyme-linked immunoassay methods to evaluate serum concentrations of infliximab and antibodies to infliximab in 32 patients with moderate to severe inflammatory bowel disease. Med. Sci. Monit..

[66] Damoiseaux J. (2020). The perspective on standardisation and harmonisation: The viewpoint of the EASI president. Autoimmun. Highlights.

[67] van Schouwenburg P.A., Kruithof S., Wolbink G., Wouters D., Rispens T. (2016). Using monoclonal antibodies as an international standard for the measurement of anti-adalimumab antibodies. J. Pharm. Biomed. Anal..

[68] Jain D., Pido M.T.J., Delgado J.C., Willrich M.A.V., Lazar-Molnar E. (2023). Comparison of two clinical laboratory assays for measuring serum adalimumab and antibodies to adalimumab. J. Appl. Lab. Med..

[69] Laserna-Mendieta E.J., Salvador-Martín S., Marín-Jiménez I., Menchén L.A., López-Cauce B., López-Fernández L.A., Lucendo A.J. (2021). Comparison of a new rapid method for determination of serum anti-adalimumab and anti-infliximab antibodies with two established elisa kits. J. Pharm. Biomed. Anal..

[70] Sam M.J., Connor S.J., Ng W.W.-S., Toong C.M.-L. (2020). Comparative evaluation of 4 commercially available elisa kits for measuring adalimumab and anti-adalimumab antibodies. Ther. Drug Monit..

[71] Song S., Yang L., Trepicchio W.L., Wyant T. (2016). Understanding the supersensitive anti-drug antibody assay: Unexpected high anti-drug antibody incidence and its clinical relevance. J. Immunol. Res..

[72] Gress K., Bass J.A., Funk R.S., Morrow R.P., Hasenkamp R., Shakhnovich V. (2020). Facing real-world challenges of immunogenicity in pediatric inflammatory bowel disease. Front. Immunol..

[73] Alghamdi A., Alahmari M., Aljohani K., Alanazi A., Al Ibrahim B., Alshowair M., Tawfik M., Alghamdi W., Alanazi S., Alzayed F. (2025). Prevalence and clinical implications of anti-drug antibody formation and serum drug levels among patients with ibd receiving anti-tnf therapy: A cross-sectional study. Saudi J. Gastroenterol..

[74] Baert F., Noman M., Vermeire S., Van Assche G., D’Haens G., Carbonez A., Rutgeerts P. (2003). Influence of immunogenicity on the long-term efficacy of infliximab in crohn’s disease. N. Engl. J. Med..

[75] Cheifetz A.S., Abreu M.T., Afif W., Cross R.K., Dubinsky M.C., Loftus E.V., Osterman M.T., Saroufim A., Siegel C.A., Yarur A.J. (2021). A comprehensive literature review and expert consensus statement on therapeutic drug monitoring of biologics in inflammatory bowel disease. Am. J. Gastroenterol..

[76] Ungar B., Chowers Y., Yavzori M., Picard O., Fudim E., Har-Noy O., Kopylov U., Eliakim R., Ben-Horin S. (2014). The temporal evolution of antidrug antibodies in patients with inflammatory bowel disease treated with infliximab. Gut.

[77] Mitrev N., Vande Casteele N., Seow C.H., Andrews J.M., Connor S.J., Moore G.T., Barclay M., Begun J., Bryant R., Chan W. (2017). Review article: Consensus statements on therapeutic drug monitoring of anti-tumour necrosis factor therapy in inflammatory bowel diseases. Aliment. Pharmacol. Ther..

