# Supplementary material for: Validation of the i-Tracker Drug and Total Anti-Drug Antibody CLIA Assays on IDS-iSYS for Therapeutic Drug Monitoring in Adalimumab- and Infliximab-Treated Patients
Source: Diagnostics (Basel). 2025 Sep 25;15(19):2447. doi: 10.3390/diagnostics15192447 (PMC12523426; doi:10.3390/diagnostics15192447)
Supplement: Supplementary file 1 [file diagnostics-15-02447-s001.zip › diagnostics-3844236-supplementary.pdf]

diagnostics

MDPI

Supplementary Materials

Validating the i-Tracker Drug and Total Anti-Drug CLIA Assays on the IDS-iSYS for Therapeutic Drug Monitoring in Adalimumab- and Infliximab-Treated Patients

Akpedje Serena Dossou *, Serena Kang, Tahira Kalhoro, Eduardo Castro-Echeverry and Nathan C. Horton *,

Sonic Reference Laboratory, Austin, TX 78728, USA
*Correspondence: adossou@sonicreferencelab.com (A.S.D.); nhorton@sonicreferencelab.com (N.C.H.)

Table S1. Reference numbers of Biosynex-Theradiag kits used in the study. The IMMUNO-TROL i-Tracker samples are external controls associated with the assays.

Kits	Biosynex-Theradiag REF#
i-Tracker Adalimumab RUO	CTA 002-100-R
i-Tracker anti-Adalimumab Total Ab RUO	CTA 003T-50-R
i-Tracker Infliximab RUO	CTI 002-100-R
i-Tracker anti-Infliximab Total Ab RUO	CTI 003T-50-R
IMMUNO-TROL i-Tracker Adalimumab	CTA 003-PC-R
IMMUNO-TROL i-Tracker anti-Adalimumab Total Ab	CTA 003T-PC-R
IMMUNO-TROL i-Tracker Infliximab	CTI 003-PC-R
IMMUNO-TROL i-Tracker anti-Infliximab Total Ab	CTI 003T-PC-R

.

 https://doi.org/10.3390/10.3390/diagnostics15192447
